# Supplementary material for: Worker ants promote outbreeding by transporting young queens to alien nests
Source: Commun Biol. 2021 May 3;4:515. doi: 10.1038/s42003-021-02016-1 (PMC8093424; doi:10.1038/s42003-021-02016-1)
Supplement: Supplementary file 2 — Supplementary Information [file 42003_2021_2016_MOESM2_ESM.pdf]

Worker ants promote outbreeding by transporting young queens to alien nests

Mathilde. Vidal[1*], Florian. Königseder[1], Julia. Giehr[1], Alexandra. Schrempf[1], Christophe. Lucas[2], Jürgen. Heinze[1]

[1] Chair of Zoology and Evolutionary Biology – University of Regensburg, Regensburg, Germany.

[2] Institut de Recherche sur la Biologie de l'Insecte (UMR7261), CNRS – Université de Tours, Tours, France.

[*]**Corresponding author and lead contact**:

Mathilde Vidal: mathilde.vidal@biologie.uni-regensburg.de

Supplementary table 1: Primer microsatellites used for the ant *Cardiocondyla elegans* with: primer sequences, repeat motif, primer pair-specific annealing temperature (TA), size range in base pair, observed number of alleles, and fluorescent primer labels.

Locus name	Primer sequences (5′to 3′)	Repeat type	TA (°C)	Size range (bp)	No. of alleles	Label of F-primer
**CE2–3A**	F: CCGTCTTTTCCACTCAC<br>R: GGAATCGTCGAGAGAGA	(AG)	60	100–134	15	TET
**CE2–4A**	F: TGCGAGTGGATGTATGA<br>R: CCCACCTTACAGCAATATC	(AG)	60	176–324	13	FAM
**CE2–5D**	F: AGACGTAAGGTTTGAAGAGA<br>R: ACAACTATGCCAAATTAAGTAT	(AC)	60	202–310	5	HEX
**CE2–12D**	F: TCCGCTAAATTATCATGG<br>R: TCGAGTGCATAAAGGAATA	(AG)	60	129–143	8	FAM
**CE2–4E**	F: ATACAAAAGAATATGAAGTAATACA<br>R: GTGTGCTTATGTATCTGGTAT	(AC)	50	136–178	19	HEX
**Card 8**	F:TCGCCGTCTATTCTGTCGTTA<br>R: CTATTATCGGCAATGTGC	(AC)	54	118–132	5	FAM
**Cobs 13**	F:TATCTTTTCAACCCTCTCGC<br>R: TATTCCGCGATAGCTTAAAT	(CT)	60	75-83	4	TET
