# Supplementary material for: Worker ants promote outbreeding by transporting young queens to alien nests
Source: Commun Biol. 2021 May 3;4:515. doi: 10.1038/s42003-021-02016-1 (PMC8093424; doi:10.1038/s42003-021-02016-1)
Supplement: Supplementary file 3 — Description of Additional Supplementary Files [file 42003_2021_2016_MOESM3_ESM.pdf]

Description of Additional Supplementary Files

File name: Supplementary Video 1

Description: (Copyright: Julia Giehr)

Movie of carrier worker going back and forth between source and recipient nests.

Time 00’06 a worker is carrying a female sexual (gyne) from a source nest.

Time 01’30 the carrier worker is dropping the gyne inside a recipient nest.

Times 01’30-3’08 the carrier worker is going back to its original source nest.

Time 03’08 the carrier worker is entering the source nest.

Time 03’16 the carrier worker (the same?) is leaving the source nest with another gyne.

Time 04’40 the carrier worker is dropping the new gyne inside the same recipient nest.

Figshare link:

https://figshare.com/s/921e9f2184716780cf0d

DOI:

10.6084/m9.figshare.12777569
