# Supplementary material for: Worker ants promote outbreeding by transporting young queens to alien nests
Source: Commun Biol. 2021 May 3;4:515. doi: 10.1038/s42003-021-02016-1 (PMC8093424; doi:10.1038/s42003-021-02016-1)
Supplement: Supplementary file 5 — Reporting Summary [file 42003_2021_2016_MOESM5_ESM.pdf]

nature research

Corresponding author(s): Mathilde Vidal

Last updated by author(s): Mar 15, 2021

Reporting Summary

Nature Research wishes to improve the reproducibility of the work that we publish. This form provides structure for consistency and transparency in reporting. For further information on Nature Research policies, see our Editorial Policies and the Editorial Policy Checklist.

Statistics

For all statistical analyses, confirm that the following items are present in the figure legend, table legend, main text, or Methods section.

n/a	Confirmed	
☐	☒	The exact sample size (n) for each experimental group/condition, given as a discrete number and unit of measurement
☐	☒	A statement on whether measurements were taken from distinct samples or whether the same sample was measured repeatedly
☐	☒	The statistical test(s) used AND whether they are one- or two-sided<br>*Only common tests should be described solely by name; describe more complex techniques in the Methods section.*
☐	☒	A description of all covariates tested
☐	☒	A description of any assumptions or corrections, such as tests of normality and adjustment for multiple comparisons
☐	☒	A full description of the statistical parameters including central tendency (e.g. means) or other basic estimates (e.g. regression coefficient) AND variation (e.g. standard deviation) or associated estimates of uncertainty (e.g. confidence intervals)
☐	☒	For null hypothesis testing, the test statistic (e.g. F, t, r) with confidence intervals, effect sizes, degrees of freedom and P value noted<br>*Give P values as exact values whenever suitable.*
☒	☐	For Bayesian analysis, information on the choice of priors and Markov chain Monte Carlo settings
☒	☐	For hierarchical and complex designs, identification of the appropriate level for tests and full reporting of outcomes
☒	☐	Estimates of effect sizes (e.g. Cohen's d, Pearson's r), indicating how they were calculated

Our web collection on statistics for biologists contains articles on many of the points above.

Software and code

Policy information about availability of computer code

Data collection	Allele size was determined using GeneScan® 500 TAMRA dye size standard and GeneScan® 3.1 software (Applied Biosystems).
Data analysis	Genetic data were analyzed using the software package GDA 34. Relatedness was estimated following Queller and Goodnight (1989) using the softwares Relatedness v4.2 and GenAlEx v6.51b2 with standard errors of means obtained by jackknifing by groups. Confidence limits for the fixation coefficients were obtained by bootstrapping over loci (5000x). From fixation coefficients F, we calculated the proportion of sib-mating α using F= α / (4–3 α) 37,38. The Mantel test was made using R software-4.0.3. Other statistical tests were conducted using Statistica v6.0 (StatSoft, Tulsa, OK).

For manuscripts utilizing custom algorithms or software that are central to the research but not yet described in published literature, software must be made available to editors and reviewers. We strongly encourage code deposition in a community repository (e.g. GitHub). See the Nature Research guidelines for submitting code & software for further information.

Data

Policy information about availability of data

All manuscripts must include a data availability statement. This statement should provide the following information, where applicable:

- Accession codes, unique identifiers, or web links for publicly available datasets
- A list of figures that have associated raw data
- A description of any restrictions on data availability

The allele data, R code for the Mantel test, all mapped colonies, data of dug colonies and pairs of gynes and carriers observed during the field trips are available in Figshare with the following link:
https://figshare.com/s/d69c30086b062b268793
DOI: 10.6084/m9.figshare.12777662

Field-specific reporting

Please select the one below that is the best fit for your research. If you are not sure, read the appropriate sections before making your selection.

☐ Life sciences ☐ Behavioural & social sciences ☒ Ecological, evolutionary & environmental sciences

For a reference copy of the document with all sections, see nature.com/documents/nr-reporting-summary-flat.pdf

Ecological, evolutionary & environmental sciences study design

All studies must disclose on these points even when the disclosure is negative.

Study description	we observed carrying of gynes by workers in the ant Cardiocondyla elegans and obtained relatedness estimates from genetic markers
Research sample	Cardiocondyla elegans
Sampling strategy	behavioral observations of gyne / worker pairs in the field, random collection
Data collection	The allele size of the microsatellites were found after the DNA extraction, sequencing and data collection of the ants. In 2014 it was made by J.Giehr, in the year 2015 by F.Königseder and A.Schrempf, and in 2018 by M.Vidal.<br>The numbers of queen carrying and colonies was respectively observed or dug in the field by J.Giehr in 2014, by F.Königseder and A.Schrempf in 2015 and 2016 and by M.Vidal in 2017, 2018 and 2019.<br>The presence or absence of sperm in the spermathecae was given by the ant dissections of A.Schrempf.
Timing and spatial scale	The lack of data for 2016 and especially 2017 is due to unsuccesfull field collection.
Data exclusions	No data were excluded from the manuscript.
Reproducibility	All attempts to repeat the experiment were successful.
Randomization	all pairs of gynes for which source and recipient colony were identified were genotyped, hence there was no need for randomization
Blinding	gynes and workers can easily be recognized by eye so it was not possible to conduct blind analyses.

Did the study involve field work? ☒ Yes ☐ No

Field work, collection and transport

Field conditions	Cardiocondyla elegans ants nest in sandy areas, nest to river banks. The production of sexual individuals usually occurs during the summer when the temperatures can variate between approximately 20 to 40°C. The ant collection or field observation, required the researchers to be bent over in their knees, looking closely at the ground for several cumulative minutes, repetitively.
Location	In seven sites in Languedoc-Roussillon (Southern France), between Beaucaire and Remoulins (BN: N 43° 50' 38.1", E 4° 36' 59.5"; CP: N 43° 51’ 9.9", E 4° 37’ 2.4"; FK: N 43° 55’ 39.8", E 4° 34’ 18.1"; H: N 43° 55’ 2.7", E 4° 35’ 4.2"; P: N 43° 56' 31.0", E 4° 33' 34.5"; RFRK: N 43° 55' 43.9", E 4° 34' 5.1"; SM: N 43° 51' 10.5", E 4° 37' 2.2"). All sites are sparsely vegetated, sandy areas on the banks of rivers Gardon and Rhône, except for site “P”, which is an unpaved sandy parking lot near the city center of Remoulins.
Access & import/export	We could reach the collection sites via car from Regensburg (Germany) to Beaucaire (France). We obtained an INTERNATIONALLY RECOGNIZED CERTIFICATE OF COMPLIANCE (IRCC) from the Access and Benefit-Sharing Clearing-House (ABSCH) for our studies and collection of the Cardiocondyla elegans ant in Gard (South of France),<br>see link: https://absch.cbd.int/database/ABSCH-IRCC-FR-247226
Disturbance	The collection of ant workers out of the nest was made by mouth vaccum aspiration. For each location, and each year, there were always a consistent number of non-disturbed nests to prevent from population extinction. When it was sufficient we only collected ant workers or carried future queens out of the nest with the help of a mouth vaccum that did not disturb the any other part of the environnement.

Reporting for specific materials, systems and methods

We require information from authors about some types of materials, experimental systems and methods used in many studies. Here, indicate whether each material, system or method listed is relevant to your study. If you are not sure if a list item applies to your research, read the appropriate section before selecting a response.

Materials & experimental systems

n/a	Involved in the study
☒	☐ Antibodies
☒	☐ Eukaryotic cell lines
☒	☐ Palaeontology and archaeology
☐	☒ Animals and other organisms
☒	☐ Human research participants
☒	☐ Clinical data
☒	☐ Dual use research of concern

Methods

n/a	Involved in the study
☒	☐ ChIP-seq
☒	☐ Flow cytometry
☒	☐ MRI-based neuroimaging

Animals and other organisms

Policy information about studies involving animals; ARRIVE guidelines recommended for reporting animal research

Laboratory animals	The study did not involve laboratory animals.
Wild animals	We observed and captured workers, queens and males of the ant species named Cardiocondyla elegans. The ants were caught using a mouth vaccum outside the nest or after having dug the nest to look for intranidal individuals. Some ants where immediately killed and stored in alcohol 100% for future microsatellites studies and others were kept in tubes provided with honey, water and cookies for the end of the field collection (1 to 4 weeks). The ants kept alive were brought to the laboratory in Regensburg and placed in vertical plaster nests moisturised and given honey, water and drosophilia to eat twice a week for later studies.
Field-collected samples	The samples from the field collection were stored in alcohol 100% for microsatellites analyses or frozen at -20°C for spermatheca dissection. We did not use live samples collected from the field in this study.
Ethics oversight	no ethical approval was required for this study

Note that full information on the approval of the study protocol must also be provided in the manuscript.
